# Skeletal muscle cellular metabolism in older HIV‐infected men

**DOI:** 10.14814/phy2.12794

**Published:** 2016-05-10

**Authors:** Heidi K. Ortmeyer, Alice S. Ryan, Charlene Hafer‐Macko, KrisAnn K. Oursler

**Affiliations:** ^1^ Department of MedicineDivision of Gerontology and Geriatric MedicineUniversity of Maryland School of MedicineBaltimoreMaryland; ^2^ Baltimore Veterans Affairs Medical Center Geriatric Research, Education, and Clinical CenterVeterans Affairs Maryland Health Care SystemBaltimoreMaryland; ^3^ Veterans Affairs Research ServiceVeterans Affairs Maryland Health Care SystemBaltimoreMaryland; ^4^ Departments of NeurologyUniversity of Maryland School of MedicineBaltimoreMaryland; ^5^ Department of Physical Therapy Rehabilitation ScienceUniversity of Maryland School of MedicineBaltimoreMaryland

**Keywords:** Cardiolipin, citrate synthase, glycogen synthase, hydrogen peroxide, *β*‐hydroxy acyl‐CoA dehydrogenase

## Abstract

Skeletal muscle mitochondrial dysfunction may contribute to low aerobic capacity. We previously reported 40% lower aerobic capacity in HIV‐infected men compared to noninfected age‐matched men. The objective of this study was to compare skeletal muscle mitochondrial enzyme activities in HIV‐infected men on antiretroviral therapy (55 ± 1 years of age, *n* = 10 African American men) with age‐matched controls (55 ± 1 years of age, *n* = 8 Caucasian men), and determine their relationship with aerobic capacity. Activity assays for mitochondrial function including enzymes involved in fatty acid activation and oxidation, and oxidative phosphorylation, were performed in homogenates prepared from vastus lateralis muscle. Hydrogen peroxide (H_2_O_2_), cardiolipin, and oxidized cardiolipin were also measured. *β*‐hydroxy acyl‐CoA dehydrogenase (*β*‐HAD) (38%) and citrate synthase (77%) activities were significantly lower, and H_2_O_2_ (1.4‐fold) and oxidized cardiolipin (1.8‐fold) were significantly higher in HIV‐infected men. VO_2_peak (mL/kg FFM/min) was 33% lower in HIV‐infected men and was directly related to *β*‐HAD and citrate synthase activity and inversely related to H_2_O_2_ and oxidized cardiolipin. Older HIV‐infected men have reduced oxidative enzyme activity and increased oxidative stress compared to age‐matched controls. Further research is crucial to determine whether an increase in aerobic capacity by exercise training will be sufficient to restore mitochondrial function in older HIV‐infected individuals.

## Introduction

The advent of highly active antiretroviral therapy (HAART) has improved survival (>35 years) for HIV‐infected people (Lohse et al. 2007) and has significantly contributed to the increase in number of older individuals (≥50 years) living with HIV (Brooks et al. 2012). However, HAART has been implicated in accelerated mitochondrial aging (Payne et al. 2011), which could adversely affect exercise tolerance. In middle‐aged (35 ± 2 years) HIV‐infected adults on HAART, low peripheral muscle oxygen extraction‐utilization (a‐vO_2_) appears to be a key reason for low aerobic capacity, compared to HIV‐infected adults not on HAART and noninfected controls, matched for age, gender and physical activity level (Cade et al. 2003). We found aerobic exercise capacity (VO_2_peak) to be 40% lower in older (52 ± 1 years) HIV‐infected men on HAART compared to healthy age‐matched men; a 45 year old HIV‐infected man had a comparable aerobic capacity of a 65‐year‐old non‐HIV‐infected man (Oursler et al. 2006). In older adults without HIV, increased age is associated with skeletal muscle mitochondrial dysfunction, which strongly correlates with low aerobic capacity (Short et al. 2005) and slower walking speed (Coen et al. 2013).

Commonly used classes of antiretroviral medications include the nucleoside reverse transcriptase inhibitors (NRTIs), protease inhibitors, and non‐nucleoside reverse transcriptase inhibitors (NNRTIs). NRTIs, like zidovudine (AZT), have long been associated with mitochondrial dysfunction, in part due to depletion of mitochondrial DNA (mtDNA), which encodes 13 polypeptides including subunits of Complex I, III, IV, and V of the electron transport chain. NRTIs decrease cytochrome *c* oxidase (COX, Complex IV) activity in cultured muscle cells prepared from human muscle biopsies (Benbrik et al. 1997). An immuno‐histochemistry study of skeletal muscle from middle‐aged adults (<50 years) shows that NRTI‐treated HIV‐infected adults have COX deficiency compared to HIV‐infected/no NRTI adults, and noninfected adults (Payne et al. 2011). The frequency of COX‐deficient muscle fibers in the NRTI‐treated middle‐aged individuals is considered similar to expected levels in healthy older adults. Protease inhibitors also cause mitochondrial dysfunction in vitro. C2C12 murine skeletal muscle cells exposed to combined protease inhibitors have reduced fatty acid oxidation, CD36 and carnitine palmitoyltransferase‐1 protein expression (Richmond et al. 1801). The combined effects of HAART and increased age on skeletal muscle mitochondrial function in HIV‐infected adults are not known.

The purpose of our study was to investigate skeletal muscle cellular metabolism, specifically activities of key enzymes involved in mitochondrial fatty acid oxidation, oxidative capacity, oxidative phosphorylation, and markers of oxidative stress in older HIV‐infected men without lipodystrophy syndrome, and to determine whether reduced mitochondrial function was associated with reduced aerobic capacity in these individuals. Since HAART is now universally recommended for HIV‐infected individuals, we chose age‐matched men without HIV as our comparison group. We hypothesized that older HIV‐infected men on HAART would have lower activities of skeletal muscle mitochondrial enzymes involved in fatty acid activation, oxidation, and oxidative phosphorylation compared to age‐matched men without HIV, and that these markers of mitochondrial function would be related to aerobic capacity. We further hypothesized that cardiolipin, a phospholipid located to the mitochondrial inner membrane and a marker of mitochondrial content (Ritov et al. 2006), would be lower in HIV‐infected men, whereas oxidized cardiolipin, which has been associated with reduced Complex I activity and accelerated aging in rodents (Petrosillo et al. 2009), and H_2_O_2_, a marker of oxidative stress, would be higher in HIV‐infected men.

## Patients & Methods

### Study population and design

This cross‐sectional study included baseline data from 10 HIV‐infected men and eight noninfected men who were enrolled in two exercise intervention trials that included muscle biopsies and were conducted in the same research unit. Participants in a trial of HIV‐infected men were matched by gender, age, and BMI to participants in a trial of overweight and obese (BMI > 25 kg/m^2^) men without HIV infection. HIV‐infected participants were recruited from a clinic‐based cohort at the Baltimore VA Medical Center and were all men and predominantly African American race. Uninfected participants were recruited from the community and were predominantly Caucasian race; therefore, it was not feasible to match by race. However, exclusion criteria for both studies were similar and included poorly controlled hypertension (systolic pressure > 180 mmHg or diastolic pressure > 105 mmHg), severe anemia (hemoglobin <10 gm/dL), and chronic renal and liver disease. Medication use, including lipid lowering agents, was not an exclusion criterion in either trial. Exercise activity (strength training or aerobic exercise more than 1 time or >20 min weekly) and history of muscle disease were exclusion criteria in both trials. Exclusion criteria for the HIV trial also included any AIDS defining condition (CDC, 1992) in the prior 6 months. Inclusion criteria unique to the HIV trial was a stable HAART regimen (same medications for ≥6 months) and age 50 years and older. Inclusion criteria unique to the trial without HIV was a BMI > 25 kg/m^2^. Both HIV‐infected and uninfected participants were sedentary (<20 min of aerobic exercise 2 times per week) prior to enrollment. Written informed consent was obtained from all subjects. Studies were approved by the University of Maryland, Baltimore Institutional Review Board and the Baltimore VA Medical Center Research and Development Committee.

### Fitness and body composition

Cardiorespiratory fitness was assessed by measuring oxygen consumption during maximum exercise effort on a treadmill (ACSM, 2014). Work rate was increased by speed and grade until the subject reached voluntary exhaustion, maximum aerobic capacity, or safety criteria defined by the American College of Sports Medicine (ACSM, 2014). VO_2_peak was measured by indirect calorimetry (Quark, Cosmed USA, Chicago, IL) and defined as the highest oxygen consumption value obtained in the last 30‐sec increment.

Subjects had fat mass, lean tissue mass, and bone mineral content determined by dual‐energy X‐ray absorptiometry (DXA) (Prodigy, LUNAR Radiation Corp., Madison, WI). Fat‐free mass (FFM) was defined as the sum of lean tissue mass and bone mass. Mid‐thigh muscle cross‐sectional area and subcutaneous fat area were determined from a single mid‐thigh computed tomography (CT) slice; abdominal visceral fat (VAT) and subcutaneous abdominal fat were determined at a single abdominal slice at the lumbar spine L4‐L5 level (Medical Imaging Processing Analysis and Visualization software (MIPAV), version 7.0.0). Muscle attenuation (intramuscular fat) by CT was measured in Hounsfield units (Ryan and Harduarsingh‐Permaul 2014).

### Laboratory testing

Testing was performed in the morning after a 12‐h overnight fast. Blood samples were drawn for the measurement of plasma glucose, insulin, and lipids. Blood samples were collected in heparinized syringes, placed in prechilled test tubes containing 1.5 mg EDTA/mL of blood, centrifuged at 4°C and stored at −80°C until analysis. Plasma glucose concentrations were measured in duplicate using the glucose oxidase method (2300 STAT Plus, YSI, Yellow Springs, OH). Plasma insulin was measured in duplicate by radioimmunoassay (Millipore Inc., St. Charles, MO). Homeostasis model assessment (HOMA) of insulin resistance (HOMA‐IR) was calculated [(fasting insulin (*μ*U/mL) × fasting glucose (mmol/L))/22.5] as described by Matthews (Matthews et al. 1985). Plasma triglyceride (TG) and cholesterol were analyzed by enzymatic methods (UniCel DxC880, Beckman Coulter, Inc., Brea, CA), and high‐density lipoprotein cholesterol (HDL‐C) was measured in the supernatant after precipitation with dextran sulfate. Low‐density lipoprotein (LDL‐C) was calculated using the Friedewald equation: LDL‐C = total cholesterol − (TG/5 + HDL‐C) (Friedewald et al. 1972). Plasma HIV viral load was measured by Abbott Real Time HIV‐1 Assay (linear range 40 to 10 × 10^6^ copies/mL).

### Muscle collection

Skeletal muscle biopsy of the vastus lateralis muscle was performed under local anesthesia (1% xylocaine) with a 5 mm Bergström needle (Stille‐Werner) with participants in a fasted state (>12 h). Muscle specimens were frozen immediately in tongs precooled by liquid nitrogen and stored at −70°C until lyophilization.

### Sample preparation and enzyme activity assays

The mitochondrial enzyme activities for each individual were measured from the same whole homogenate sample. Eleven milligrams of lyophilized microdissected skeletal muscle were homogenized in 330 *μ*L of buffer (1:30) containing (in mmol/L) 250 sucrose, 10 Tris•HCl pH 7.4, 1 EDTA, and protease inhibitors (Roche 11836170001). Several aliquots of the 1:30 homogenate were frozen in liquid nitrogen and stored at −80°C until assay for NADH dehydrogenase (NADH‐D), succinate dehydrogenase (SDH), coenzyme Q:cytochrome *c* – oxidoreductase (Complex III), COX, and ATP synthase activities. Thirty microliter of the 1:30 homogenate was diluted to 1:150 in homogenization buffer, frozen in liquid nitrogen and stored at −80°C until assay for citrate synthase, *β*‐hydroxyacyl CoA (*β*‐HAD), acyl CoA synthase (ACS), and total protein.

ACS activity was measured as described (Mashek et al. 2006) using 10 *μ*L of the 1:150 homogenate in 190 *μ*L of assay cocktail. The reaction was terminated after 10 min at 37°C by the addition of 1 mL Dole's reagent (isopropanol:heptane:1N H2SO4, 80:20:2. v/v). The labeled substrate for this assay was [1‐^14^C]palmitic acid. The labeled product ([^14^C]‐acyl‐CoA) was measured by scintillation counting following two 2 mL heptane washes.

Carnitine palmitoyltransferase (CPT‐1) activity was measured as described (Brown 2003) using 10 *μ*L of the 1:30 fresh homogenate in 10 *μ*L 5X substrate cocktail, 25 *μ*L 2X assay cocktail and either 5 *μ*L homogenization buffer or 5 *μ*L 100 μmol/L malonyl‐CoA. The final concentration of palmitoyl‐CoA was 300 μmol/L. The reaction was terminated after 10 min at 30°C by the addition of 0.5 mL 1.2 N HCl. The labeled substrate for this assay was L‐[N‐methyl‐^14^C]carnitineHCl. The labeled product (palmitoyl‐[^14^C]carnitine) was measured by scintillation counting following the addition of 500 *μ*L 1‐butanol.

*β*‐HAD and citrate synthase activities were measured by continuous spectrophotometric rate determination as described (Lynen and Wieland 1955; Srere 1969) using 10 *μ*L of the 1:150 homogenate in a final volume of 1 mL.

NADH‐D, SDH, COX, and ATP synthase activity were measured in microplate assay kits (MitoSciences MS141, MS241, MS443, MS543, respectively) using 3–10.5 *μ*L of the 1:150 homogenate per well.

Complex III activity was measured as described (Luo et al. 2008) using 5 *μ*L of the 1:30 homogenate in a final volume of 200 *μ*L that included (final concentration in mmol/L) 0.05 cytochrome c, 50 Tris‐HCl pH 7.4, 4 NaN3, 0.05 decylubiquinol, and 0.05% Tween‐20 in the absence and presence of antimycin A (10 μmol/L).

Catalase, glutathione peroxidase (GPx), and superoxide dismutase (SOD) enzyme activities for each individual were measured from the same supernatant. Five milligrams of lyophilized microdissected skeletal muscle was homogenized in 150 *μ*L of buffer (1:30) containing (in mmol/L) 250 sucrose, 10 Tris•HCl pH 7.4, 1 EDTA, and protease inhibitors (Roche 11836170001). The homogenate was centrifuged at 10,000 × *g* for 15 min then several aliquots of the 1:30 supernatant were frozen in liquid nitrogen and stored at −80°C until assay. For catalase (abcam ab83464, fluorometric method) and GPx (abcam ab102530, colorimetric method), the 1:30 sample was diluted fourfold with ice‐cold assay buffer. Diluted supernatant of 4 *μ*L was used in the catalase assay (sample and sample high control) and 10 *μ*L in the GPx assay. For SOD (abcam ab65354, colorimetric method), 12 *μ*L of the undiluted supernatant was used.

ATP and intramuscular triglyceride (IMTG): Five milligrams of lyophilized microdissected skeletal muscle was homogenized in 375 *μ*L of cell lysis reagent (Roche). Whole homogenate of 25* μ*L was removed to determine ATP content as described (Wang et al. 2011). The sample for ATP was diluted 10‐fold in cell lysis reagent before undergoing six freeze–thaw cycles (liquid nitrogen to room temperature) followed by boiling for 15 min to inactivate ATPase activity. The sample was centrifuged at 17,900 × *g* for 5 min and the supernatant further diluted 10‐fold in cell lysis reagent. The ATP Bioluminescence Assay Kit HS II (Roche) and a SpectraMax L luminescence microplate reader (Molecular Devices) were used to determine ATP levels. Lipids were extracted (Bligh and Dyer 1959) from the remaining homogenate (350 *μ*L) and solubilized in PBS containing 5% Triton X‐100. IMTG was determined using the EnzyChrom Triglyceride Assay Kit (BioAssay Systems).

Hydrogen peroxide (H_2_O_2_), glycogen synthase (GS), glucose 6‐phosphate (G6P), and glycogen: Two milligrams of lyophilized microdissected skeletal muscle was homogenized in 300 *μ*L of buffer containing (in mmol/L) 50 tricine pH 7.4, 100 NaF, 10 EDTA, and protease inhibitors (Roche 11836170001). The homogenate was centrifuged at 13,000 × *g* for 5 min at 4°C. An aliquot of supernatant was removed for GS activity and total protein determination and the remaining supernatant was frozen and boiled for 5 min, and centrifugation repeated. This last supernatant was used for fluorometric determination of H_2_O_2_ using the Hydrogen Peroxide Assay Kit (abcam), G6P using the High Sensitivity Glucose‐6‐Phosphate Assay Kit (Sigma), and glycogen using the Glycogen Assay Kit (Sigma). GS activity was measured as previously described (Ryan et al. 2012).

### Cardiolipin

Lipids were extracted from 1 mg microdissected lyophilized skeletal muscle using chloroform/methanol. Cardiolipin (18:2)_4_ MW 1448 was separated and characterized by reverse‐phase ion pair high‐performance liquid chromatography‐mass spectrometry by the Center for Mitochondrial Diseases, Case Western Reserve University, Cleveland, OH as described (Minkler and Hoppel 2010). The oxidized molecular species of cardiolipin (1‐O_2_ MW 1464 and 2‐O_2_ MW 1480) were further characterized as described (Kim et al. 2011).

All mitochondrial enzyme activities, ATP, and H_2_O_2_ were expressed per *μ*g cardiolipin and per mg total protein (Coomassie Plus, Pierce). Catalase, GPx, SOD, IMTG, GS, G6P, and glycogen were expressed per mg total protein. The homogenates and supernatants prepared from the HIV‐infected and control individuals were run with the same substrate for all assays. Each sample was run in triplicate with the median value used for data analysis.

### Statistics

Comparisons of the HIV groups were performed using unpaired Student's t‐tests for GS, G6P, and glycogen. Pearson correlation was used to examine the relationship between mitochondrial function and aerobic capacity. VO_2_peak (L/min) was tested with adjustment for total body weight (mL/kg/min) and FFM (mL/kg FFM/min). Variables with significant linear relationships were further tested using linear regression models that included a potential confounding variable. To test the independent association of HIV with the dependent variables for mitochondrial function and aerobic capacity, the following potential confounders were included in a step‐wise fashion in the models with HIV: age, adiposity (% body fat), statin use, and insulin use. Statistical significance was set at a two‐tailed *P* < 0.05.

## Results

### Subjects

HIV‐infected participants were receiving HAART that included at least one NRTI medication [Tenofovir (Broholm et al. 2013), Abacavir (Broholm et al. 2013), or AZT (Benbrik et al. 1997)] and was combined with either a protease inhibitor with Ritonavir (*n* = 7) or Efavirenz (*n* = 3). The median (IQR) CD4 cell count was 422 cells/mL (345‐627); none of the participants had a CD4 cell count below the threshold for definition of AIDS (<200 cells/mL) nor an AIDS defining condition (CDC, 1992) for at least 6 months prior to enrollment. All the participants had HIV‐1 level below the level of detection (<40 c/mL). Except for higher triglycerides in the control subjects (*P* = 0.05), there were no differences in fasting HDL‐C, LDL‐C, insulin, or glucose values between groups (Table 1). None of the participants had lipodystrophy syndrome as defined by fat mass ratio (Freitas et al. 2010). However, two of the HIV‐infected participants were receiving insulin for diabetes mellitus; therefore their data were excluded from fasting insulin and HOMA‐IR analyses. VO_2_peak (mL/kg/min) and VO_2_peak (mL/kg FFM/min) were 24% and 33% lower in HIV‐infected men compared to controls, respectively (*P* < 0.01 and *P* < 0.0002) (Table 1). All individuals had VO_2_peak categorized as very poor (*n* = 10), poor (*n* = 5), or fair (*n* = 3) based on the Fitness Categories for Maximal Aerobic Power for Men by Age (ACSM, 2014).

**Table 1:** Subject characteristics

Variables (mean ± SE)	HIV‐infected *N* = 10	Controls *N* = 8
Age (years)	55 ± 1 (51–62)	55 ± 1 (45–64)
BMI (kg/m^2^)	27 ± 1 (21–35)	29 ± 1 (26–33)
Weight (kg)	81 ± 5	93 ± 4
Waist circumference (cm)	89 ± 3^b^	103 ± 3
VO_2_peak (L/min)	1.9 ± 0.2^c^	3.1 ± 0.2
VO_2_peak (mL/kg/min)	25 ± 3^a^	33 ± 1
VO_2_peak (mL/kg FFM/min)	33 ± 2^d^	49 ± 2
Whole Body DXA		
Fat‐free mass (kg)	56 ± 2	63 ± 2
Fat mass (kg)	22 ± 3^a^	31 ± 2
Body fat (%)	26 ± 3	32 ± 1
Abdominal and mid‐thigh CT Scans^e^		
Visceral fat area, VAT (cm^2^)	100 ± 18^a^	158 ± 19
Subcutaneous fat area, SAT (cm^2^)	241 ± 45	310 ± 22
VAT/SAT ratio	0.43 ± 0.06	0.51 ± 0.05
Mid‐thigh muscle area (cm^2^)	116.3 ± 6.3	118.3 ± 7.6
Mid‐thigh subcutaneous fat area (cm^2^)	59.2 ± 9.5	63.5 ± 8.1
Intramuscular fat (HU)	42 ± 2	40 ± 2
Serum Lipids^f^		
Total Cholesterol (mg/dL)	162 ± 14	188 ± 12
HDL‐C (mg/dL)	50 ± 7	42 ± 3
LDL‐C (mg/dL)	94 ± 10	109 ± 12
Triglycerides (mg/dL)	113 ± 1^a^	187 ± 37
Fasting glucose (mmol/L)	5.7 ± 0.4	5.6 ± 0.3
Fasting insulin^g^ (pmol/L)	105 ± 27	92 ± 12
HOMA IR^g^	4.1 ± 1.1	3.9 ± 0.7

^a^ *P* ≤ 0.05; ^b^ *P* < 0.01; ^c^ *P* < 0.001; ^d^ *P *< 0.0002 compared to controls.

^e^HIV‐infected *n* = 9.

^f^Statins used in 4 HIV‐infected subjects and 1 control subject.

^g^Excluded data from two HIV‐infected subjects on insulin.

Details on anthropometric measures, DXA and CT scan results by group are summarized in Table 1. There were no significant differences in fat‐free mass, mid‐thigh muscle, subcutaneous fat area, and intramuscular fat between groups. Despite matching by BMI, HIV‐infected men had lower total fat mass and percent body fat compared to controls. The difference may be driven by central obesity; greater central abdominal fat by VAT and waist circumference were found in the control group.

### Skeletal muscle

**Figure 1:**
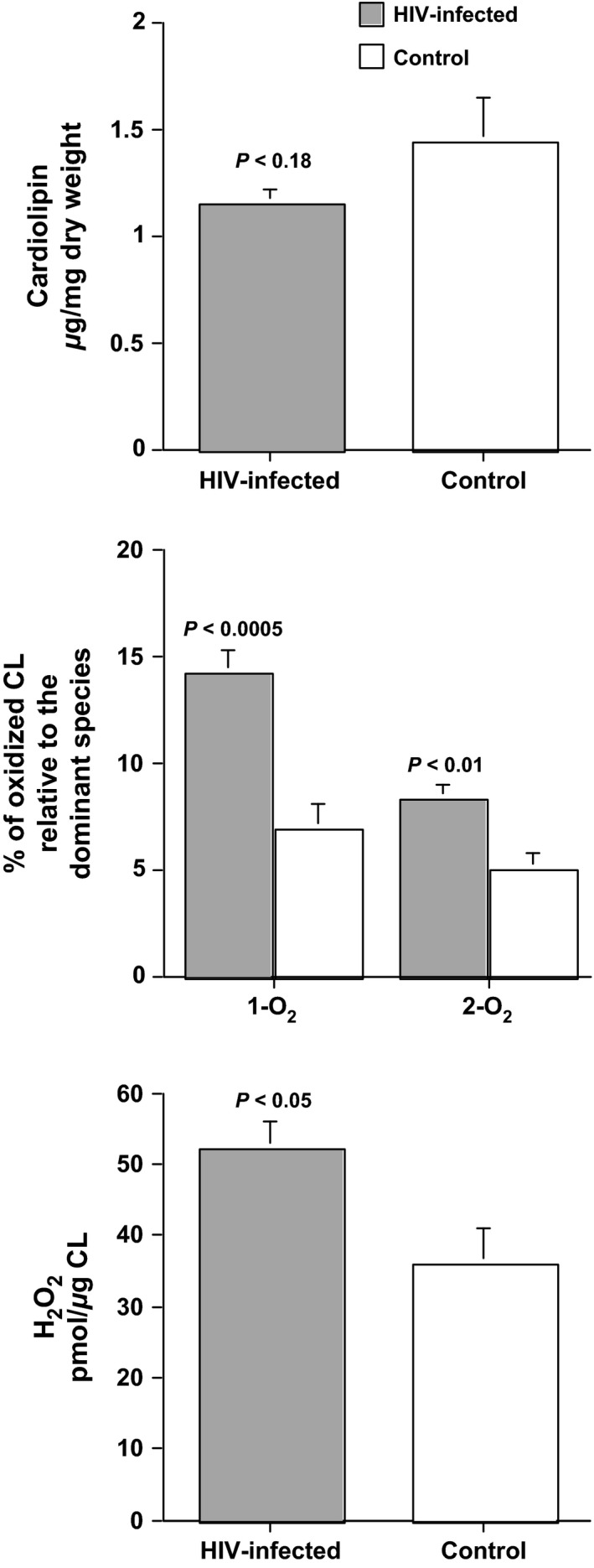
Cardiolipin (CL), oxidixed CL, and hydrogen peroxide (H_2_O_2_) in skeletal muscle samples from HIV‐infected men (*n* = 10) and age‐matched controls (*n* = 8). Upper panel: CL content is not significantly different between the two groups. Middle panel: Oxidized CL (one oxygen, 1‐O_2_; two oxygens, 2‐O_2_) is significantly lower in HIV‐infected men (*n* = 10) versus age‐matched controls (*n* = 8). Lower panel: H_2_O_2_ is significantly higher in HIV‐infected men (*n* = 8) versus age‐matched controls (*n* = 8).

**Figure 2:**
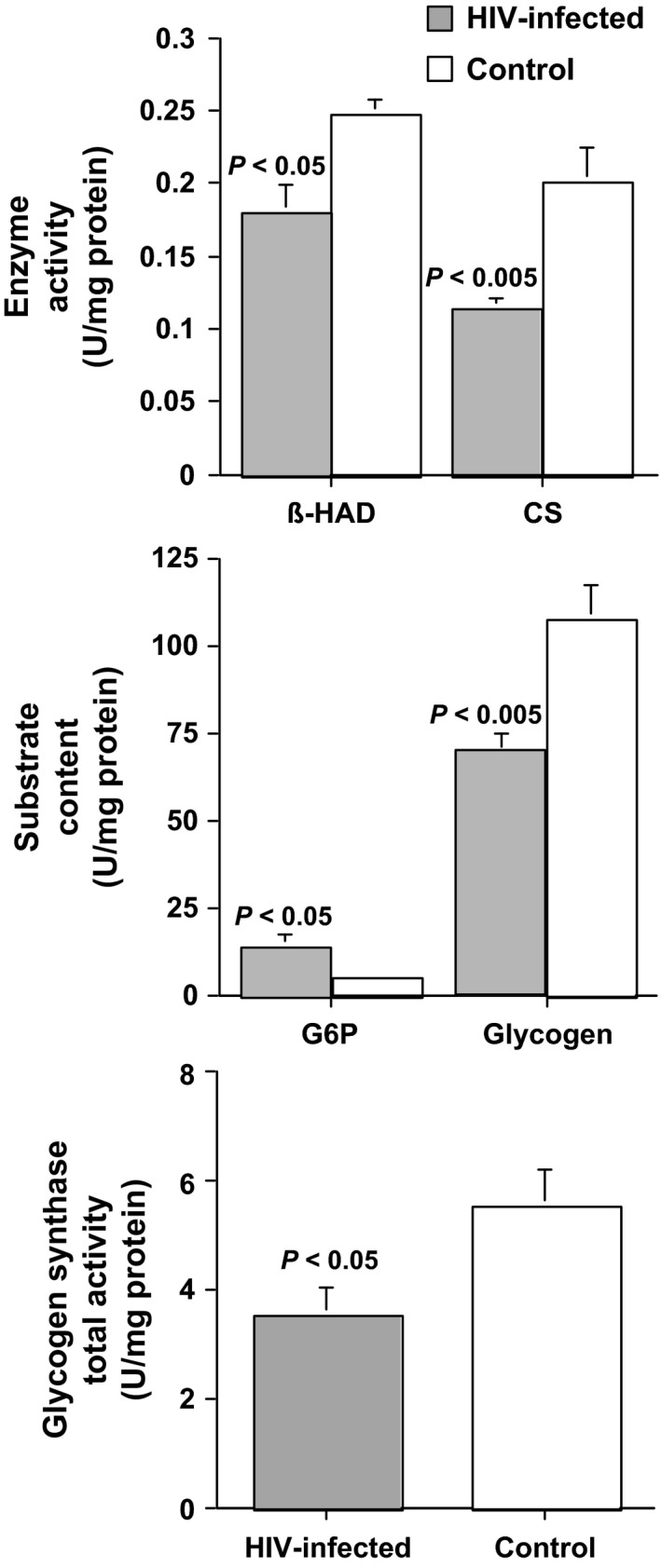
Enzyme activity and substrate content in HIV‐infected men versus age‐matched controls. Upper panel: *β*‐hydroxyacyl CoA dehydrogenase (*β*‐HAD) and citrate synthase (CS) activities are significantly lower in HIV‐infected (*n* = 10) versus age‐matched controls (*n* = 8). Middle panel: Glucose‐6‐phosphate (G6P) is higher, whereas glycogen is lower in HIV‐infected men (*n* = 8) compared to age‐matched controls (*n* = 8). Lower panel: Glycogen synthase (GS) total activity is significantly lower in HIV‐infected individuals (*n* = 8).

Cardiolipin content was 20% lower in the HIV‐infected individuals compared to control subjects but was not significantly different between groups (*P* = 0.17) (Fig. 1). The oxidized species of cardiolipin were twofold (1‐O_2_) and 1.6‐fold (2‐O_2_) higher in the HIV‐infected individuals (Fig. 1). Results for both species of oxidized cardiolpin were unchanged in adjusted models that included HIV status and each of the possible confounding factors (age, percent body fat, statin use, and insulin use). The results for both species of oxidized cardiolipin were confirmed by repeat analysis of 10 of the original 18 samples. The data between each run were strongly correlated (1‐O_2_, *r* = 0.93, *P* < 0.001; 2‐O_2_, *r* = 0.94, *P* < 0.001). H_2_O_2_ content was 1.4‐fold higher in the HIV‐infected men (Fig. 1). H_2_O_2_ content remained significantly different between the HIV groups in the model adjusted for age but lost significance when adjusted for percent body fat, statin use, and insulin use. G6P was threefold higher in the HIV‐infected men and glycogen was 36% lower in HIV‐infected men (Fig. 2). Neither ATP nor IMTG were different between the groups (Table 2).

**Table 2:** Enzyme Activity and Substrate Content (mean ± SE)

	U/*μ*g cardiolipin		U/mg total protein	
	HIV‐infected (*n* = 10)	Controls (*n* = 8)	HIV‐infected (*n* = 10)	Controls (*n* = 8)
Acyl‐CoA synthase	147 ± 27	153 ± 41	1017 ± 113	1132 ± 237
Carnitine palmitoyltransferase	0.76 ± 0.14	0.66 ± 0.20	5.2 ± 0.6	5.0 ± 1.4
*β*‐hydroxyacyl CoA dehydrogenase	23.7 ± 2.6†	34.6 ± 5.6	Figure 2, *	Figure 2
Citrate synthase	16.3 ± 2.5*	25.7 ± 3.2	Figure 2, **	Figure 2
NADH dehydrogenase(Complex I)	74 ± 10	72 ± 12	545 ± 58	505 ± 34
Succinate dehydrogenase(Complex II)	0.67 ± 0.11	1.1 ± 0.42	4.8 ± 0.6	6.6 ± 1.7
Coenzyme Q:cytochrome *c*oxidoreductase (Complex III)	0.32 ± 0.03	0.29 ± 0.05	2.9 ± 0.3	3.2 ± 0.7
Cytochrome *c* oxidase(Complex IV)	0.85 ± 0.11	0.93 ± 0.17	6.2 ± 0.6	6.6 ± 0.6
ATP synthase (Complex V)	2.2 ± 0.16	2.2 ± 0.29	17 ± 2	15 ± 0.7
Catalase	na	na	1.4 ± 0.5	1.8 ± 0.4
Glutathione peroxidase	na	na	62 ± 11	80 ± 42
Superoxide dismutase	na	na	7.8 ± 3	6.1 ± 3
H_2_O_2_	Figure 1, *	Figure 1	1114 ± 108*	781 ± 86
ATP	42 ± 4	48 ± 5	72 ± 6	87 ± 10
Intramuscular triglyceride	na	na	14 ± 2	13 ± 2

Different than age‐matched controls, ^†^ *P* = 0.06, *P < 0.05, **P < 0.005.

*β*‐HAD (38%) and citrate synthase (77%) activities were significantly lower in HIV‐infected individuals versus controls (Fig. 2). Both activities remained significantly different by HIV group in models adjusted for a potential confounding variable (age, percent body fat, statin use, insulin use). There were no significant differences in the following enzyme activities between HIV‐infected men and controls, ACS, CPT‐1, NADH dehydrogenase, SDH, ubiquinol‐cytochrome c reductase, COX, ATP synthase, catalase, GPx, and SOD (Table 2). Glycogen synthase total activity was 36% lower in HIV‐infected individuals versus controls (Fig. 2). Glycogen synthase independent activity (HIV vs. control, 0.11 ± 0.3 nmol/min/mg protein vs. 0.15 ± 0.3 nmol/min/mg protein) and fractional activity (3 ± 0.4% vs. 3 ± 0.7%) were not different between the groups.

**Figure 3:**
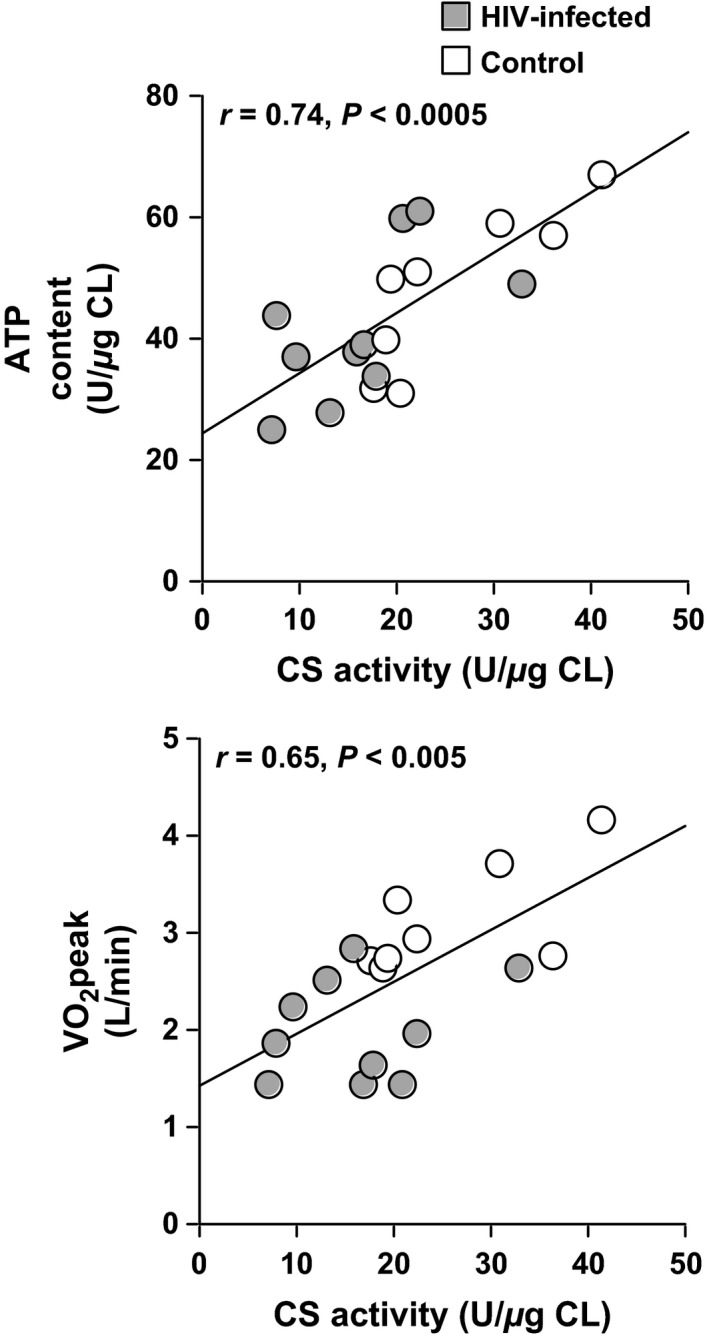
Upper panel: Skeletal muscle citrate synthase (CS) is associated with ATP content in the combined groups (*n* = 18). Lower panel: CS is associated with peakVO_2_ in the combined groups (*n* = 18).

Glycogen synthase total activity was related to glycogen content (*r* = 0.48, *P* = 0.05). H_2_O_2_ was inversely related to catalase activity (*r* = −0.50, *P* < 0.05). Cardiolipin was related to citrate synthase activity (per total protein) (*r* = 0.56, *P* < 0.05). Both oxidized species of cardiolipin (1‐O_2_ and 2‐O_2_) were inversely related to citrate synthase activity (*r* = −0.61, *P* < 0.01 and *r* = −0.58, *P* < 0.05, respectively). *β*‐HAD (*r* = 0.56, *P* < 0.05) and citrate synthase were related to ATP content (*r* = 0.74, *P* < 0.0005) (Fig. 3). Plasma triglyceride was inversely related to CPT‐1 activity (*r* = −0.61, *P* < 0.01) and COX activity (*r* = −0.49, *P* < 0.05).

*β*‐HAD (*r* = 0.65, *P* < 0.005), citrate synthase (*r* = 0.65, *P* < 0.005) (Fig. 3), H_2_O_2_ (*r* = −0.64, *P* < 0.01), and oxidized cardiolipin (1‐O_2_; *r* = −0.59, *P* < 0.01) (2‐O_2_; *r* = −0.47, *P* < 0.05) correlated with VO_2_peak (L/min). *β*‐HAD (*r* = 0.59, *P* = 0.01), citrate synthase (*r* = 0.53, *P* < 0.05), H_2_O_2_ (*r* = −0.68, *P* < 0.005), and oxidized cardiolipin (1‐O_2_; *r* = −0.52, *P* < 0.05) also correlated with VO_2_peak (mL/kg FFM/min). These results were not affected by adding a potential cofounding variable (age, percent body fat, statin use, insulin use) to the model.

## Discussion

The results of our study demonstrate that older HIV‐infected men on HAART have reduced oxidative enzyme activity (*β*‐HAD and citrate synthase) and higher oxidized cardiolipin when compared with age‐matched controls, even when controlled for the potentially confounding variables age, percent body fat, statin use, and insulin use. The significant association of H_2_O_2_ with HIV was attenuated when controlled for statin use (*P* = 0.06) and insulin use (*P* = 0.08) and lost statistical significant when controlled for percent body fat (*P* = 0.22). All four mitochondrial markers, *β*‐HAD, citrate synthase, H2O2, and oxidized cardiolipin, were related to aerobic capacity, with or without adding the potential confounding variables to the model. Therefore, reduced *β*‐HAD and citrate synthase activities, and increased oxidized cardiolipin and H_2_O_2_ are likely affected by, and/or contribute to, low aerobic exercise capacity in this population.

Studies of skeletal muscle mitochondrial enzyme activity have focused on HIV‐infected patients with clinical manifestations of medication side effects, including lipodystrophy syndrome (body fat redistribution, insulin resistance, and dyslipidemia). Mitochondrial dysfunction in an HIV‐infected person with HAART‐related lipodystrophy was first described in 2000 (Miro et al. 2000). The individual had high neutral lipids in type I and II myocytes, decreased mitochondrial activities of Complex III and IV, and multiple deletions in skeletal muscle mtDNA (Miro et al. 2000). A follow‐up study by this group compared similar variables in seven HIV‐infected people with lipodystrophy syndrome to four HIV‐infected people without lipodystrophy syndrome and 12 healthy controls (Zaera et al. 2001). Based on scatterplots, Complex I and II activities appear to be similar across all three groups, whereas Complex III and IV activities were lowest in the HIV‐infected individuals with lipodystrophy syndrome, compared to the HIV‐infected individuals without lipodystrophy syndrome and the healthy controls (Zaera et al. 2001). In our study, there were no significant differences in Complex I, II, III, IV, or V activities, or intramuscular triglyceride content, between older HIV‐infected without lipodystrophy syndrome and controls.

Cardiolipin is a unique phospholipid which is localized primarily in the inner mitochondrial membrane and is associated with enzymes in the electron transport chain (Houtkooper and Vaz 2008). Under normal conditions, cytochrome *c* is anchored to the mitochondrial membrane by cardiolipin (Fariss et al. 2005). Due to cardiolipins location near the site of oxygen radical production and its high content of unsaturated fatty acids, cardiolipin is highly susceptible to peroxidative attack by oxyradicals. Peroxidation of cardiolipin abolishes cytochrome *c* binding (Shidoji et al. 1999) which would adversely affect electron transport chain activity, especially the activities of Complex III and IV. Cardiolipin and Complex I activity were reduced in heart mitochondria in old (24 month) rats, whereas oxidized cardiolipin and H_2_O_2_ were higher in old versus young (4 month) rats (Petrosillo et al. 2009). AZT administered to mice caused an 11‐fold increase in peroxide production compared to control mice (de la Asuncion et al. 1998). In our study, the HIV‐infected men had 1.6‐ to 2‐fold higher oxidized cardiolipin and 1.4‐fold higher H_2_O_2_ compared to the age‐matched control group, suggesting accelerated mitochondrial aging and greater oxidative stress in the HIV‐infected men, although the enzyme activities of the electron transport chain were similar between the two groups. It is likely that the enzyme activities of the electron transport chain, especially Complex III and IV, were reduced in vivo in the HIV‐infected men as the in vitro assays for Complex III and IV provided saturating amounts of cytochrome c. Although not significantly different between the groups, the mean activities of glutathione peroxidase and catalase were >20% lower in the HIV‐infected men, which could collectively contribute to reduced antioxidant capacity and lead to increased peroxidation of cardiolipin. Oxidized cardiolipin and H_2_O_2_ were inversely related to VO_2_peak, and oxidized cardiolipin was inversely related to citrate synthase activity. Our results suggest that increased oxidized cardiolipin and H_2_O_2_, while not adversely affecting the intrinsic electron transport chain enzyme activity, may contribute to reduced aerobic capacity in our population of HIV‐infected men.

The effects of HIV therapy alone or HIV plus therapy on skeletal muscle ACS or CPT‐1 activity, two key enzymes involved in mitochondrial fatty acid metabolism, have not been previously reported. In vitro, protease inhibitors (ritonavir plus atazanavir, lopinavir, or darunavir) reduced 14C‐palmitate oxidation and CPT‐1 protein expression in C2C12 myotubes (Richmond et al. 1801). Although enzyme activities were not measured in the aforementioned study, decreased ACS, CPT‐1, *β*‐HAD and/or citrate synthase activity could all contribute to reduced 14C‐palmitate oxidation. In our study, neither ACS nor CPT‐1 activities were different between HIV‐infected and control subjects, suggesting that HIV plus modern therapy does not adversely affect these key enzymes of fatty acid oxidation.

Our finding of higher skeletal muscle glucose‐6‐phosphate and lower glycogen in the HIV‐infected men could be a result of reduced glycogen synthase total activity. Broholm et al. (Broholm et al. 2013) reported similar basal glycogen synthase total activity in HIV‐infected individuals with lipodystrophy compared to age‐ and VO_2_max‐matched controls. Haugaard et al. (2005a) reported similar basal glycogen synthase total activity and glycogen content between HIV‐infected individuals with and without lipodystrophy. In both these studies (Haugaard et al. 2005a; Broholm et al. 2013) the HIV‐infected individuals with lipodystrophy were more insulin‐resistant by HOMA‐IR and glucose utilization than the comparison groups, whereas in our study, the HIV‐infected individuals and the controls were equally insulin‐resistant by HOMA‐IR. However, our HIV‐infected individuals were less fit by VO_2_peak than the controls, which could explain their reduced glycogen synthase total activity.

Roge et al. conducted a study of eight HIV‐infected individuals (median age 54) with lipodystrophy syndrome and/or hyperlactemia while on HAART with a NRTI‐base of either stavudine or didanosine (Roge et al. 2002). Interestingly, despite the similarity in age and aerobic capacity of these HIV‐infected subjects to HIV‐infected subjects in our study, Roge found no significant differences in VO_2_max, citrate synthase and *β*‐HAD activities, and ATP and glycogen content between the HIV‐infected subjects and age‐matched controls (Roge et al. 2002); we found significant differences between VO_2_peak, citrate synthase, *β*‐HAD and glycogen between HIV‐infected subjects and age‐matched controls. The notable differences between the two study populations are the NRTI regimens and the body composition. None of our HIV‐infected subjects had a history of lipodystrophy syndrome. In fact, both DXA and CT data showed less adiposity in the HIV‐infected subjects compared to the uninfected subjects.

While mtDNA depletion has been associated with NRTI therapy, there may be direct adverse effects from HIV infection itself. Haugaard et al. (2005b) showed that HIV‐infected individuals who were NRTI naïve had lower skeletal muscle mtDNA compared with those treated with NRTIs. Whether mtDNA depletion is due to therapy and/or the virus, a decrease in mtDNA content could adversely affect mitochondrial function by reducing the expression and/or activity of four of the five enzymes in the mitochondrial respiratory chain encoded by mtDNA (Complex I, III, IV, and V). In our study, the intrinsic activities of all four enzymes encoded by mtDNA were remarkably similar between older men with and without HIV, suggesting similar mtDNA between the two groups. A study of HIV‐infected men receiving NRTI therapy compared to HIV‐infected men who had never been treated showed lower mtDNA and citrate synthase activity in the men receiving NRTI, but similar COX activity and COX‐II gene expression in PBMCs (Miro et al. 2004). The authors suggest that reduced citrate synthase activity is indicative of decreased mitochondrial mass and that normal mitochondrial respiratory chain complex activities and expression could be due to compensatory mechanisms at the translational or posttranslational level (Miro et al. 2004).

In a study of 13 healthy adults, Larsen et al. (Larsen et al. 2012) reported significant relationships between mitochondrial content and both cardiolipin and citrate synthase activity. This group concluded that cardiolipin is the most valid biomarker for mitochondrial content as citrate synthase is involved in mitochondrial substrate oxidation. In a study of noninfected individuals, Ritov et al. (Ritov et al. 2010) showed that cardiolipin was similar in lean and obese individuals, and tended to be lower in individuals with type 2 diabetes, whereas citrate synthase activity was higher in obese compared to the other two groups. This group also proposed that cardiolipin best reflects the surface area and accordingly the mass of inner mitochondrial membrane. In our study, citrate synthase and cardiolipin were significantly associated in the entire group; however, there was no relationship between citrate synthase activity and cardiolipin content in the HIV‐infected men (*r* = 0.03, *P* = 0.93, *n* = 10). Our results confirm that citrate synthase activity is not a suitable marker of mitochondrial content in all study populations.

Presumably, a defect in the activity of any of the mitochondrial enzymes examined in our study could lead to lower ATP production. ATP content was 17% reduced in the HIV‐infected men but not significantly different than in the control subjects; however, ATP content was related to *β*‐HAD and to citrate synthase activities suggesting that the individuals with the greatest enzyme activity had the highest ATP content. Sinwell et al. (Sinnwell et al. 1995) showed that ATP content at rest was the same in subjects with HIV with or without AZT therapy and in controls. However, steady‐state levels of phosphocreatine during graded exercise were depleted to a greater extent in the HIV subjects on AZT therapy, and modeling suggested that AZT treatment decreased the maximal rate of ATP synthesis and maximal work output (Sinnwell et al. 1995).

Limitations of our study include a small sample size and two groups that differed by race and body composition despite matching by BMI. Furthermore, the HIV‐infected subjects were more likely to have history of diabetes and statin use compared to the control group. We acknowledge these important potential confounding factors. Lower aerobic capacity in the HIV‐infected individuals was unlikely due to race, as 40–49‐year‐old non‐Hispanic black (*n* = 89) and non‐Hispanic white (*n* = 227) men have similar estimated VO_2_max (Wang et al. 2010). In a previous study from our group, neither VO_2_max nor citrate synthase activity were different between African American (33.8 mL/kg FFM/min, 0.083 U/mg protein) and Caucasian (30.7 mL/kgFFM/min, 0.084 U/mg protein) men (Ryan et al. 2014). The difference in H_2_O_2_ was also unlikely due to race, as Caucasian men and women have higher plasma H_2_O_2_ production compared to African American men and women (Lacy et al. 2000). Statin use in 4/10 HIV‐infected subjects compared to 1/8 control subject, and insulin use in 2/10 HIV‐infected subjects could have biased mitochondrial enzyme activities to be lower and markers of oxidative stress higher in the HIV‐infected group. However, the statistically significant differences in *β*‐HAD activity, citrate synthase activity, and oxidized cardiolipin between the two groups remained even after controlling for potential confounding variables (age, adiposity, statin use, and insulin use). Only H_2_O_2_ lost statistical significance when controlling for adiposity, statin use (from *P* = 0.03 to *P* = 0.06), and insulin use. Nevertheless, our findings support the need for a larger study with carefully selected comparison groups to further investigate the cellular mechanisms(s) for lower mitochondrial function and aerobic capacity in HIV‐infected adults.

In summary, older HIV‐infected men on antiretroviral therapy have reduced oxidative (*β*‐HAD and citrate synthase) enzyme activity and increased markers of oxidative stress (H_2_O_2_ and oxidized cardiolipin), all of which are significantly associated with low aerobic capacity. Further research is essential to determine what aerobic exercise training prescription, if any, will be sufficient to delay mitochondrial aging and restore mitochondrial function in older HIV‐infected individuals.

## Conflict of Interest

The authors have no conflict of interest to declare.
